# Imidacloprid Induces Neurotoxicity in Albino Male Rats by Inhibiting Acetylcholinesterase Activity, Altering Antioxidant Status, and Primary DNA Damage

**DOI:** 10.1155/2023/4267469

**Published:** 2023-09-11

**Authors:** Hossam El Din H. Abdelhafez, Fatma M. Hammam, Asmaa A. EL-Dahshan, Hussien AboDalam, Jiangfeng Guo

**Affiliations:** ^1^Mammalian and Aquatic Toxicology Department, Central Agricultural Pesticides Laboratory, Agricultural Research Center, P.O. Box. 12618, Dokki, Giza, Egypt; ^2^Department of Zoology, Faculty of Science (Girls Branch), Al-Azhar University, Cairo, Egypt; ^3^Plant Pathology Department, Faculty of Agriculture, Cairo University, Giza, Egypt; ^4^College of Life Sciences and Medicine, Zhejiang Sci-Tech University, Hangzhou 310018, Zhejiang, China

## Abstract

Imidacloprid (IMI) is a neonicotinoid insecticide used worldwide, either alone or in combination with other pesticides. The goal of this study was to assess the effects of IMI on the central nervous system of rats and its mechanism of oxidative stress-induced DNA damage by oxidant/antioxidant parameters. Fifteen male rats, divided into three groups, were used: the first group received 5 ml/kg body weight corn oil as a control, the second received a high oral dose of IMI (45 mg/kg body weight), while the third received a low dose (22 mg/kg body weight). After 28 days, acetylcholinesterase (AChE) activity, oxidative stress markers, histopathological alterations, and DNA damage were examined in the brains of these rats. The AChE activities decreased significantly after IMI exposure, reaching 2.45 and 2.75 nmol/min/mg protein in high dose and low dose, respectively, compared to the control group (3.75 nmol/g tissues), while the concentration of malondialdehyde MDA increased significantly (29.28 and 23.92 nmol/g tissues) vs. the control group (19.28 nmol/g tissues). The antioxidant status parameters such as reduced glutathione (GSH) content was 13.77 and 17.63 nmol/g, catalase (CAT) activity was 22.56 and 26.65 *µ*mol/min/g, and superoxide dismutase (SOD) activity was 6.66 and 7.23 *µ*mol/min/g in both doses against the control group (21.37 nmol/g, 30.67 *µ*mol/min/g, 11.76 *µ*mol/min/g), respectively, and histopathological changes in the brain tissues were observed. More in vivo research using epigenetic methods is needed to determine the ability of IMI and its metabolites to cause neurotoxicity and DNA lesions in mammalian brains.

## 1. Introduction

Neonicotinoids, which first appeared in the late 1980s, now constitute one-third of the global pesticide market and are rapidly replacing organophosphates, carbamates, and pyrethroids [1]. Nicotine is a part of their structure, and they specifically act on nicotinic acetylcholine receptors [2]. Imidacloprid (IMI) is an agonist nicotinic acetylcholine receptor that is very effective against a variety of sucking insects [3, 4]. Its action causes an in acetylcholine, which paralyzes the insect and eventually kills it [3]. Inhibition of the enzyme acetylcholinesterase (AChE) was one of the first biomarkers identified for human exposure to environmental pesticides. Guerra et al. found a statistically significant decrease in zebrafish brain AChE activity after being exposed to 15 and 45 *µ*g/L IMI for 96 hours [5]. On the other hand, Sevim et al. discovered no significant differences in AChE levels between IMI-exposed and control L-929 fibroblasts [6].

Oxidative stress and lipid peroxidation (LPO) are considered good biomarkers for assessing insecticide-induced hazardous effects in numerous organisms [7–9]. Oxidative damage harms biological systems by contributing to lipid, DNA, and protein damage, leading to cell death. Numerous studies have shown that IMI causes oxidative stress and lipid peroxidation in mammals and cell lines [10, 11]. Orally exposing female rats to 20 mg/kg IMI resulted in a significant elevation of malondialdehyde (MDA) levels and a decrease of redox parameters such as reduced glutathione (GSH), catalase (CAT), and superoxide dismutase (SOD) in their livers, kidneys, and brains [12]. Furthermore, IMI disrupts compound ion channels or neurotransmission, leading to irreversible neurotoxicity in mammals which may eventually devolve into chronic neurodegenerative disorder.

Humans are primarily exposed to IMI through drinking water and food. Concerningly, IMI can cause carcinogenic and mutagenic effects in both animals and humans [13]. The first stage of genotoxicity is DNA damage, which disrupts biological structures and functions, and causes reproductive and carcinogenic problems [14]. DNA oxidation is a useful marker for determining mutagenicity mechanisms [15]. IMI's mutagenic potential has been demonstrated in vitro and in vivo using genomic endpoints, for instance, the micronucleus test, chromosomal aberrations, the comet assay, and DNA damage [16–18]. Moreover, exposure occurs in the nutrition through imported agricultural goods, till now [19]. The aim of this research was to discover the mechanism of neurotoxicity by which IMI affects cholinesterase activity, antioxidant status biomarkers, and primary DNA damage in rat brains after a 28-day oral dosage.

## 2. Materials and Methods

### 2.1. Chemicals

IMI (95.45% pure) was obtained from Central Agricultural Pesticides Lab. (CAPL), Agricultural Research Center, Dokki, Egypt. Sigma Chemical Co. (USA) supplied all the other chemicals used in this study. Prior to each experiment, all lab solutions were freshly prepared.

### 2.2. Animals

For 2 weeks, all 15 adult male rats (*Rattus norvegicus*, weighing about 200 ± 20 g, National Research Centre, Giza, Egypt) were acclimatized prior to the treatment by housing them under standard conditions of 50–55% humidity, 22°C ± 2°C, and 12 ± 1 h of light/dark cycles, with open access to water and consumed with commercial pelleted diet (obtained from Modern Mills Company, Giza, Egypt). The Cairo University Animal Ethics Committee approved the animal guidelines used in this study (Permission No. CU/III/F/5/20) according to with (EU Directive 2010/63/EU).

### 2.3. Experimental Design

The IMI doses were chosen based on the documented median lethal dose value: 450 mg/kg body weight [20]. The time period of the experiments was varied according to the results of the subacute study conducted by Test No. 407 [21]. IMI was dissolved in corn oil and administered orally daily for 4 weeks. The animals were divided randomly into three groups (*n* = 5 per group) and gavage-treated for 28 days at 45 mg/kg B.W. IMI for high dose and 22.5 mg/kg B.W. IMI for low dose whilst the control group received 5 ml/kg B.W. with corn oil as vehicle.

### 2.4. Euthanasia and Sample Collection

After 28 days, the animals were anaesthetized prior to cervical dislocation with intraperitoneal injections of ketamine (90 mg/kg B.W.) and xylazine (5 mg/kg B.W.) [22]. Brain samples were quickly extracted, rinsed in ice-cold saline, homogenized, and used to determine biochemical parameters. The brain homogenates were centrifuged at 14000 rpm for 20 minutes at 4°C (MSE Super-Minor centrifuge, England), and the supernatants were collected to measure LPO levels (MDA), GSH contents, antioxidant enzyme activities (CAT and SOD), and AChE activity using the V-670 UV/VIS/NIR spectrophotometer (190–2700 nm wavelength). Brian tissue samples were also dissected for examining DNA fragmentation and their histopathology.

### 2.5. Acetylcholinesterase Activity

AChE activity was evaluated using a previously described method by the authors in [23]. The reaction mixture, containing 2.6 ml of sodium phosphate buffer (0.1 M, pH 7.5), 0.15 ml of dithiobis (2-nitrobenzoic acid) (DTNB, 10 mM, pH 7.0, containing 3 mg of NaHCO_3_ per 8 mg of DTNB), and 0.1 g of the supernatant, was kept at room temperature for 10 min. The reaction was started by adding 0.15 ml of acetylthiocholine iodide (12.5 mM), and the absorbance change per minute was measured at 412 nm for 4 min. Specific activity is expressed in nmol·min^−1^·mg^−1^ protein.

### 2.6. Lipid Peroxidation Level

MDA, a byproduct of LPO, is commonly detected by the thiobarbituric acid reactive substances (TBARS) assay because it reacts with TBARS to produce a fluorescent product [24]. Thiobarbituric acid (TBA) reacts with MDA in an acidic medium for 30 min at 95°C to form a TBAR product, which can be measured at 534 nm. MDA levels are expressed in nmol g^−1^ tissue.

### 2.7. Oxidative Stress Markers

GSH content was assessed by the method described in [25], CAT activity was estimated using a previously described method [26], and SOD activity was measured using the autoxidation and illumination of pyrogallol at 440 nm for 3 min 40, according to Marklund and Marklund [27].

### 2.8. Total DNA Fragmentation

DNA fragmentation was qualitatively assessed by agarose gel electrophoresis through a DNA laddering assay [28, 29]. To clarify, the brain tissue was homogenised in a hypotonic lysis buffer, the lysates were centrifuged for 10 minutes, and the supernatants containing small DNA fragments were separated from the intact DNA pellets. The DNA was resuspended in Tris-ethylenediaminetetraacetic acid (Tris-EDTA) buffer for electrophoretic analysis on a 2% agarose gel. Under ultraviolet light, the gel was stained with ethidium bromide and observed.

### 2.9. Histopathological Examination

Brain cells were collected from all groups, immersed in 10% formol-saline for 24 h, embedded in paraffin wax, sectioned at 4 mm, and stained with hematoxylin and eosin. The slides were examined under a microscope to assess pathomorphological alterations in the brain tissues [30].

### 2.10. Statistical Analysis

The data are displayed as mean ± standard deviation (M ± SD). The one-way ANOVA (SPSS, SPSS Inc., Chicago, IL, USA) version 17 and GraphPad Prism, version 8 software (San Diego, CA), compared the groups. A *p* value of <0.05 is recorded.

## 3. Results and Discussion

### 3.1. Acetylcholinesterase Activity

**Figure 1:**
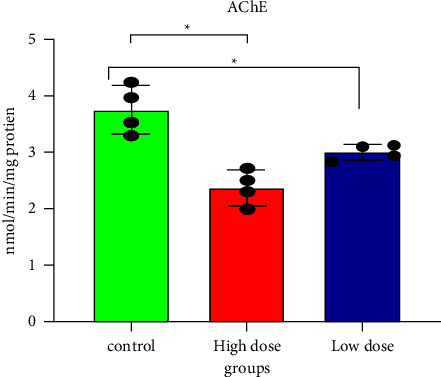
Acetylcholinesterase activity in rat brain tissues after 28 days of oral IMI exposure. Values are expressed as the mean ± SD expressed as nmol of acetylthiocholine hydrolyzed per minute per mg protein. 45 mg/kg B.W., and 22.5 groups vs. control *p* < 0.05 three groups (*n* = 5).

The nicotinergic neurons in the brain are inactivated by IMI, a chloronicotinyl insecticide used systemically [31, 32]. As shown in Figure 1 and Table 1, all investigated doses of IMI significantly reduced AChE activity in rat brains after 28 days, compared with the control (*p* < 0.05), eventually leading to neurotoxicity. IMI binds, either with high affinity or partially, to specific subsites on nicotinic acetylcholine receptors (nAChRs), stimulating them and inducing neurotoxicity [19, 33, 34]. This continuous stimulation affects both nervous function and AChE activity [35]. Kimura-Kuroda et al. showed that exposing a Sprague–Dawley rat cerebellar cell line to IMI-induced conformational changes in the Ach receptors and enhanced cellular Ca^2+^ uptake via *α*7 nAChRs [32]. Increased total cholinesterase activity, which reduces ACh-assisted IMI binding, may be an adaptive response to oxidative stress caused by increased Ca^2+^ uptake [36]. Like our results, Topal et al. reported decreased AChE activity in the brains of fish exposed to IMI [37].

### 3.2. Lipid Peroxidation

**Figure 2:**
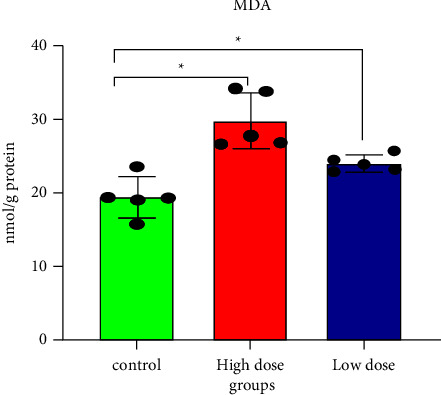
Malondialdehyde levels in rat brain tissues after 28 days of oral IMI exposure. Values are expressed as the mean ± SD expressed as nmol of MDA per g protein. 45 mg/kg B.W., and 22.5 groups vs. control *p* < 0.05 three groups (*n* = 5).

Increased free radicals generated during IMI exposure, combined with the decreased capacity of the cell to scavenge them, may be responsible for free radical-induced membrane lipid peroxidation (MDA) in rat tissues [10].  Figure 2 and Table 1 show that MDA significantly increased in brain tissues following both the oral doses of IMI, compared with the control. A high concentration of polyunsaturated fatty acids in biological systems, combined with a low antioxidant capacity, makes them more susceptible to LPO and oxidative stress [38]. Furthermore, IMI metabolites, such as desnitro metabolites, and nitromethylene analogs are more dangerous to mammals [19, 31, 39]. IMI metabolites might be sources of free radicals that induce abundant amounts of MDA. Numerous findings have suggested that IMI exposure is associated with LPO in rodent livers and kidneys [12, 40].

### 3.3. Oxidative Stress Biomarkers

**Figure 3:**
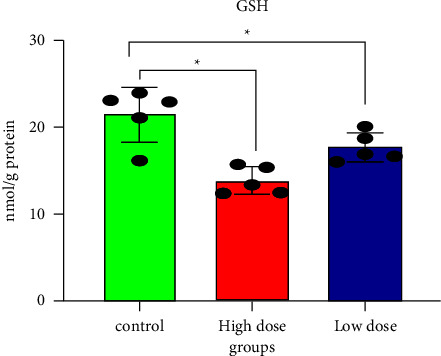
Reduced glutathione content in rat brain tissues after 28 days of oral IMI exposure. Values are expressed as the mean ± SD expressed as mmol of g tissues. 45 mg/kg B.W., and 22.5 groups vs. control *p* < 0.05 three groups (*n* = 5).

**Figure 4:**
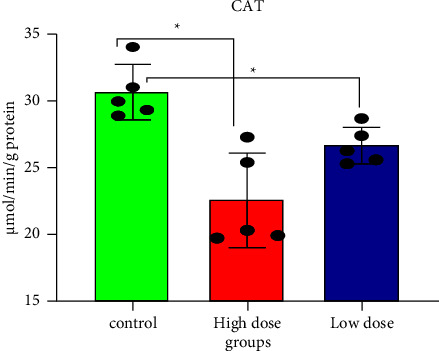
Catalase levels in rat brain tissues after 28 days of oral IMI exposure. Values are expressed as the mean ± SD expressed as units of CAT per g protein. 45 mg/kg B.W., and 22.5 groups vs. control *p* < 0.05 three groups (*n* = 5).

**Figure 5:**
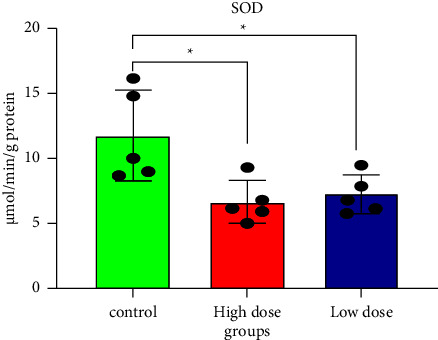
Superoxide dismutase levels in rat brain tissues after 28 days of oral IMI exposure. Values are expressed as the mean ± SD expressed as units of SOD per g protein. 45 mg/kg B.W., and 22.5 groups vs. control *p* < 0.05 three groups (*n* = 5).

Pesticide concentrations and structures may alter the efficiency of the GSH redox cycle. Furthermore, enzymes such as SOD and CAT neutralize the reactive oxygen species (ROSs) produced during pesticide exposure [10, 41]. GSH and the vitamins C and E have been shown to protect cells and tissues from xenobiotic oxidative stress [42]. Several studies have shown that IMI has oxidative and neurotoxic potential in mammals [32, 39, 43]. Figures 3–5, and Table 1 show that all doses of IMI led to a significant decrease in GSH, CAT, and SOD levels compared with the control (*p* < 0.05). IMI's metabolism is fast and metabolite accumulation in tissues may result in an excess of free radicals/ROS, which could damage mitochondrial membrane structures, having caused permeability changes as evidenced by increased LPO and GSH depletion [44]. Mahajana et al. showed that IMI exposure reduced antioxidant enzymes (SOD and CAT) responsible for scavenging superoxides, peroxides, and hydroxides generated during toxicant-induced oxidative stress in the kidney tissue [10].

### 3.4. Total DNA Fragments

**Figure 6:**
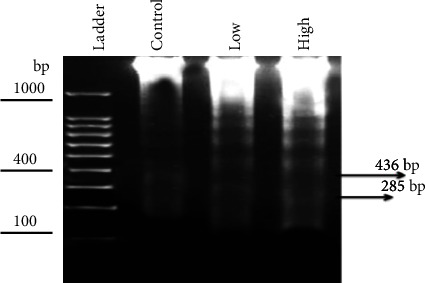
Agarose gel electrophoresis was used to evaluate the fragmentation of rat brain DNA in all experimental groups. Ladder DNA marker, DNA patterns of the control group, the treated group (low dose), and the treated group (high dose).

DNA damage can disrupt biological pathways, resulting in a genotoxic disorder linked with reproductive and carcinogenic diseases [45]. IMI's mutagenic potential has been demonstrated in in vitro and in vivo systems using cytogenetic endpoints, such as the micronucleus test, the comet assay, and the DNA fragmentation assay [13].  Figure 6 shows that brain cells frequently contained fragmented DNA, observed using gel electrophoresis, and these fragmentations were used as a damage characteristic. DNA, isolated from the brains of rats exposed to IMI at doses of 45 mg/kg and 22 mg/kg body weight, had degraded into oligonucleotide fragments, forming a clear laddering pattern when resolved by electrophoresis. The higher dose caused more DNA fragmentation, yielding four bands (790.12, 627.72, 436.2, and 258.47 bp) compared with control, while the lower dose yielded three bands (810.59, 622.61, and 439.91 bp). ROS levels increased with increasing IMI concentrations, resulting in more DNA damage [18, 46, 47]. Change in glutathione status due to toxic agents causes oxidative stress, which leads to mammalian genotoxicity [48]. IMI is an alkylating material that can cause clastogenesis by damaging cellular DNA. The presence of an electronegative group in the nitroguanidine moiety of neonicotinoids may link IMI to DNA, inducing the formation of breaks [2]. IMI-induced DNA damage could also be attributed to direct reactions with the pesticide or their metabolites, which destabilize the DNA structure and cause breaks [49].

### 3.5. Histopathological Findings

**Figure 7:**
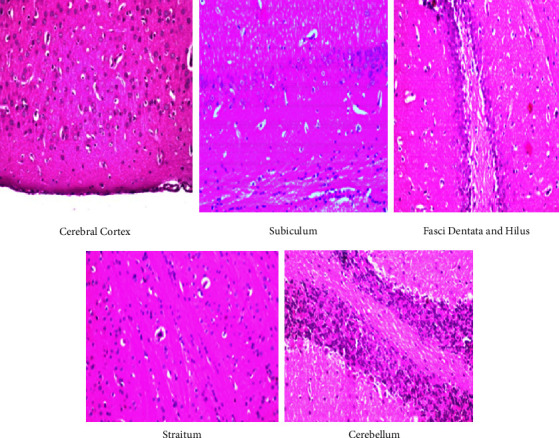
Displayed a photomicrograph of the cerebral cortex, subiculum, fasci dentata and hilus, straitum, and cerebellum in rat brain tissues for histopathological extermination in a control group.

**Figure 8:**
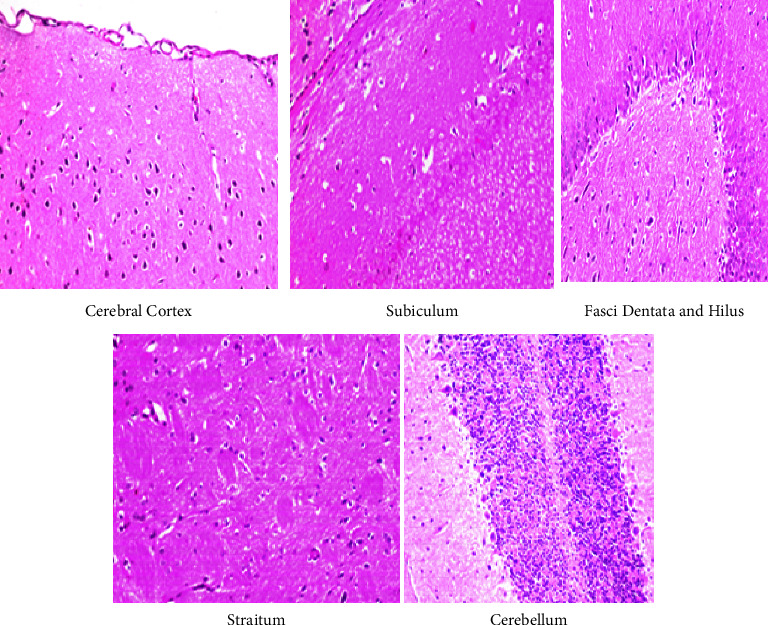
Displayed a photomicrograph of the cerebral cortex, subiculum, fasci dentata and hilus, straitum, and cerebellum in rat brain tissues for histopathological changes in a low dose group.

**Figure 9:**
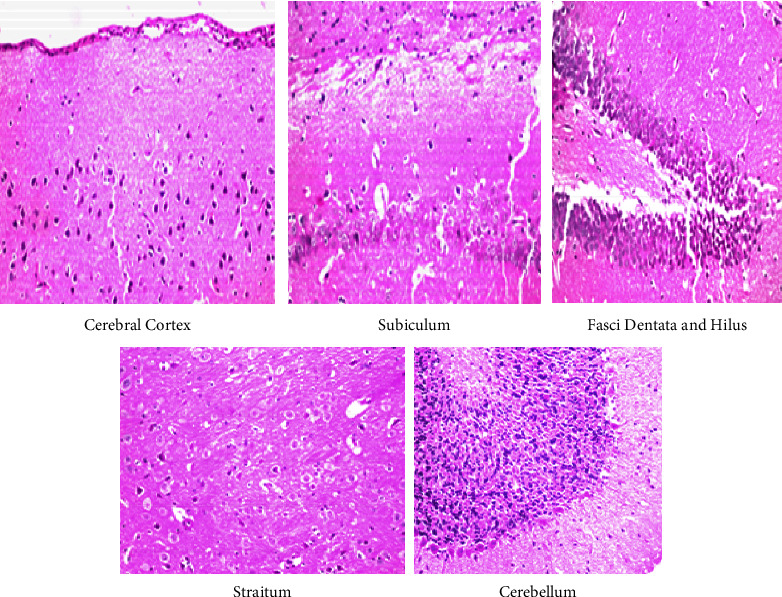
Displayed a photomicrograph of the cerebral cortex, subiculum, fasci dentata and hilus, straitum, and cerebellum in rat brain tissues for histopathological changes in a high dose group.

The histopathology of the cerebral cortex, hippocampus, striatum, and the cerebellum of control group rats was unaltered, thus showing a normal histological neuronal structure (Figure 7). Rats exposed to the lower dose of IMI showed nuclear pyknosis and degeneration in the neurons of the cerebral cortex, fascia dentata, and the hilus, with intracellular edema in the striatum, and necrosis in the granular layer of the cerebellum (Figure 8). Similar observations were made in rats administered the higher dose of IMI, along with focal eosinophilic plagues in the striatum (Figure 9). These histopathological findings are in line with the results of Abd-Elhakim et al., who found varying degrees of neuronal degeneration in the brains of male rats exposed to 1 mg/kg body weight IMI per day [50]. In addition, exposure to IMI for 28 days induced necrosis of Purkinje cells with loss of dendrites and granules in the granular layer of the cerebellum in female rats [43]. The brain of IMI-treated mice 28 (for 90 days) revealed focal neuronal degeneration, hemorrhages, necrosis, and congestion of cerebral blood vessels, besides congestion and degeneration in areas of the hippocampus [51]. IMI was detectable and caused DNA damage in the brains of male rats treated orally with it for 28 days (2.25 mg/kg body weight/day) [18]. Degenerative changes in Purkinje cells of the cerebrum have been recorded in rats exposed to high doses of IMI [52]. Also, brain sections of rats treated with IMI displayed perivascular hemorrhage and nuclear migration of neurons with degenerative changes, which provide support to the neurobehavioral effects indicating accumulation of IMI and its metabolites in the brain [53].

## 4. Conclusion

The subacute IMI exposure can cause toxic effects, which can lead to a variety of neurodegenerative diseases, depending on the dose which its outcomes AChE inhibition; changes in antioxidant status caused by increased lipid peroxidation and a decrease in GSH, CAT, and SOD. In addition, high and low doses induced genotoxicity by DNA fragmentation. The results show that IMI can decrease new neurons, through deterioration in brain cells by histopathological examination and cell membrane via increment of lipid peroxidation. Our data suggest that IMI induces oxidative stress which produces ROS leading to abnormal total DNA, moreover inhibition of acetylcholinesterase activity in rat brain. All of the abovementioned biological events have been suggested to be possible mechanisms for IMI neurotoxicity. These results provide a possible theoretical basis for evaluating the harm of IMI to mammals and environment. Thus, we can conclude from our study that the nervous system of mammals, including humans, is more sensitive to excessive consumption of IMI in the environment as a replacement for traditional insecticides, and that much more research is needed to prove how IMI and its metabolites promote oxidative stress, which causes alterations in the mRNA levels of genes that encode antioxidant proteins.

## Figures and Tables

**Table 1:** The effects of sublethal Imidacloprid doses on antioxidant parameters, lipid peroxidation, and neural biomarker (AChE) in rat brain tissues.

Treatment	AChE (ml mol/min/mg protein)	MDA (nmol/g tissues)	GSH (nmol/g tissues)	CAT (*µ*mol/min/g tissues)	SOD (*µ*mol/min/g tissues)
Control	3.75 ± 0.41	19.28 ± 2.79	21.37 ± 3.12	30.67 ± 2.08	11.76 ± 3.48
45 mg/kg IMI high dose	2.47 ± 0.41^a^	29.79 ± 3.82^a^	13.77 ± 1.58^a^	22.56 ± 3.53^a^	6.66 ± 1.65^a^
22.5 mg/kg IMI low dose	2.75 ± 0.42^a^	23.92 ± 1.18^a^	17.63 ± 1.64^a^	26.65 ± 1.4^a^	7.23 ± 1.51^a^

Data are expressed as mean ± SD (*n* = 5) ^a^Statistical significance between imidacloprid treatment groups versus control group (the one-way ANOVA, *p* < 0.05).

## Data Availability

The data used to support the study are available from the corresponding author upon request.
